# Splanchnic Artery Stenosis and Abdominal Complaints: Clinical History Is of Limited Value in Detection of Gastrointestinal Ischemia

**DOI:** 10.1007/s00268-012-1485-4

**Published:** 2012-02-22

**Authors:** R. W. F. ter Steege, H. S. Sloterdijk, R. H. Geelkerken, A. B. Huisman, J. van der Palen, J. J. Kolkman

**Affiliations:** 1 Department of Gastroenterology, University Medical Centre Groningen, Hanzeplein 1, Postbox 30001, 9700 RB Groningen, The Netherlands; 2 Department of Gastroenterology, Medical Spectrum Twente, Enschede, The Netherlands; 3 Department of Vascular Surgery, Medical Spectrum Twente, Enschede, The Netherlands; 4 Department of Radiology, Medical Spectrum Twente, Enschede, The Netherlands; 5 Department of Epidemiology, Medical Spectrum Twente, Enschede, The Netherlands; 6 Department of Research Methodology, Measurement, and Data Analysis, University of Twente, Enschede, The Netherlands

## Abstract

**Background:**

Splanchnic artery stenosis is common and mostly asymptomatic and may lead to gastrointestinal ischemia (chronic splanchnic syndrome, CSS). This study was designed to assess risk factors for CSS in the medical history of patients with splanchnic artery stenosis and whether these risk factors can be used to identify patients with high and low risk of CSS.

**Methods:**

All patients referred for suspected CSS underwent a standardized workup, including a medical history with questionnaire, duplex ultrasound, gastrointestinal tonometry, and angiography. Definitive diagnosis and treatment advice was made in a multidisciplinary team. Patients with confirmed CSS were compared with no-CSS patients.

**Results:**

A total of 270 patients (102 M, 168 F; mean age, 53 years) with splanchnic artery stenosis were analyzed, of whom 109 (40%) had CSS and 161 no CSS. CSS-patients more often reported postprandial pain (87% vs. 72%, *p* = 0.007), weight loss (85% vs. 70%, *p* = 0.006), adapted eating pattern (90% vs. 79%, *p* = 0.005) and diarrhea (35% vs. 22%, *p* = 0.023). If none of these risk factors were present, the probability of CSS was 13%; if all were present, the probability was 60%. Adapted eating pattern (odds ratio (OR) 3.1; 95% confidence interval (CI) 1.08–8.88) and diarrhea (OR 2.6; 95% CI 1.31–5.3) were statistically significant in multivariate analysis.

**Conclusions:**

In patients with splanchnic artery stenosis, the clinical history is of limited value for detection of CSS. A diagnostic test to detect ischemia is indispensable for proper selection of patients with splanchnic artery stenosis who might benefit from treatment.

## Introduction

The classical presentation of patients with chronic gastrointestinal ischemia, also called chronic splanchnic syndrome (CSS), consists of postprandial abdominal pain, weight loss, and an abdominal bruit. However, it has been shown that this classical triad is present in a minority [1–4]. In CSS, the postprandial pain typically starts 15–30 min after the meal, may last for 2–3 hours, and may result in fear of eating. The reduced caloric intake for fear of eating causes the serious weight loss. Changes in bowel habits, usually diarrhea, may sometimes occur [1, 4, 5]. Nausea, dyspepsia with fullness, bloating, and pain after mental stress or exercise also have been mentioned [6, 7]. Data on the clinical presentation depend on retrospective studies reporting on case series of 20 to 144 patients [1, 3]. To our knowledge, no prospective studies of patients with splanchnic artery stenosis comparing the clinical history in those with and without ischemia have been published. Thus, the precise role of clinical features in diagnosing CSS is unknown.

The prevalence of asymptomatic splanchnic artery stenosis increases up to 30–50% with age in elderly subjects [8, 9]. In the majority of subjects these stenosis remain asymptomatic (chronic splanchnic disease or CSD), whereas in some CSS develops. Splanchnic artery stenosis is caused by atherosclerosis in most cases. If ischemic complaints have developed in presence of extrinsic compression of the celiac artery by the arcuate ligament of the diaphragm, celiac artery compression syndrome (CACS) is diagnosed. Among young women, this is the most prevalent cause of CSS [10]. Still, most subjects with compression of the celiac artery remain asymptomatic.

We and others have demonstrated that measurement of an increased PCO_2_ in the gastric and small bowel lumen is indicative of local ischemia [11, 12]. This local PCO_2_ stems from buffering of protons produced during anaerobic metabolism in the mucosal tissue, and is present in all studied ischemia models [12]. The PCO_2_ can be measured with a nasogastric balloon-tipped catheter attached to modified capnograph, and allows for semicontinuous measurement. An abnormal tonometry test after exercise or test meals allowed for accurate selection of patients who may benefit treatment of vessels stenosis [3, 13].

Because the number of “incidentally found” splanchnic artery stenosis will increase as a result of increased use of high-resolution CT scan and MRI, the risk of overdiagnosis and overtreatment is quite conceivable. It would therefore be desirable to establish the sensitivity, specificity, and positive and negative predictive value of the various parameters in the medical history that may specifically point to the presence or absence of clinical relevant ischemia. That would allow for better identification of patients in whom diagnostic workup, such as tonometry, and treatment would be indicated and in whom it could be omitted. Our group, a nationwide referral center for splanchnic vascular disorders, has ample experience with analysis and treatment of this complex group of patients [11, 14].

During the past decade, 40–50% of patients with splanchnic artery stenosis who were referred where diagnosed with CSS [11, 15]. The purpose of this study was to assess risk factors for CSS in the medical history of patients with splanchnic artery stenoses and whether these risk factors can be used to identify patients with high and low risk of CSS.

## Patients and methods

### Patient selection and data collection

All patients referred for possible CSS undergo a standardized diagnostic workup as previously reported [13]. All data are recorded in a database. For this study, we included data of all patients referred between February 1, 2006 and May 31, 2009. Exclusion criteria were: 1) incomplete diagnostic workup (no tonometry and no imaging or patients who were not discussed in our Multi-Disciplinary Team (MDT) for splanchnic ischemia; 2) previous treatment for CSS; 3) nonobstructive mesenteric ischemia (NOMI), i.e., gastrointestinal ischemia in absence of splanchnic artery stenosis; 4) alternative diagnosis found during evaluation; 5) follow-up less than 3 months after treatment or patients lost to follow-up after treatment.

### Medical history and clinical features

All patients were asked to complete a questionnaire (see Appendix). The questions were based on our initial studies and relevant literature [3, 4, 6, 10, 11]. The medical history notes of the two experienced main investigators (JKO and RHG) also were analyzed to maximize the potential of valuable factors. Both investigators have more than 20 years of experience in the field of CSS. A clinical feature (e.g., weight loss) could be scored in both the questionnaire and medical history notes.

### Duplex ultrasound

Duplex ultrasonography (DU) was performed after 6 hours of fasting and using a standard protocol. The definition of normal and stenotic artery origins was based on the criteria published by Moneta et al. [15].

### Tonometry

The principles of air tonometry have been described previously [2, 11]. Exercise tonometry was performed before, during, and after 10 minutes of submaximal exercise. The criteria as published by Otte et al. were used to define a positive test result [11]. In the 24-hour tonometry, standardized meals were given to patients to provoke ischemia [3]. The criteria published by Mensink et al. were used to define a positive test result [16, 17].

### Angiography

Digital subtraction angiography of the splanchnic arteries was performed if the result of tonometry and/or duplex ultrasound was abnormal. The origins of the splanchnic vessels were visualized during expiration and inspiration as described previously [3].

### Diagnosis and follow-up

The results of the diagnostic workup were discussed in the MDT consisting of a gastroenterologist, a vascular surgeon, and an interventional radiologist and a consensus diagnosis was made. The *initial* diagnosis was based on 1) clinical presentation, 2) presence of splanchnic artery stenosis, and 3) results of tonometry. The diagnosis was classified as CSS, CSD, or no CSD (alternative diagnosis). In this study, the CSS group consisted of 1) CSS due to atherosclerotic stenosis and 2) CSS due to eccentric celiac artery compression by the arcuate ligament with ischemia (CACS). Only patients with the diagnosis CSS were considered for treatment.

The *final* diagnosis was based on the outcome of follow-up, including the effects of treatment. In treated patients, the diagnosis was sustained if symptoms were severely reduced or completely resolved after successful revascularization of the affected vessels. If an alternative explanation for the complaints was found, the diagnosis was adjusted. The final diagnosis was used for the analysis.

**Fig. 1:**
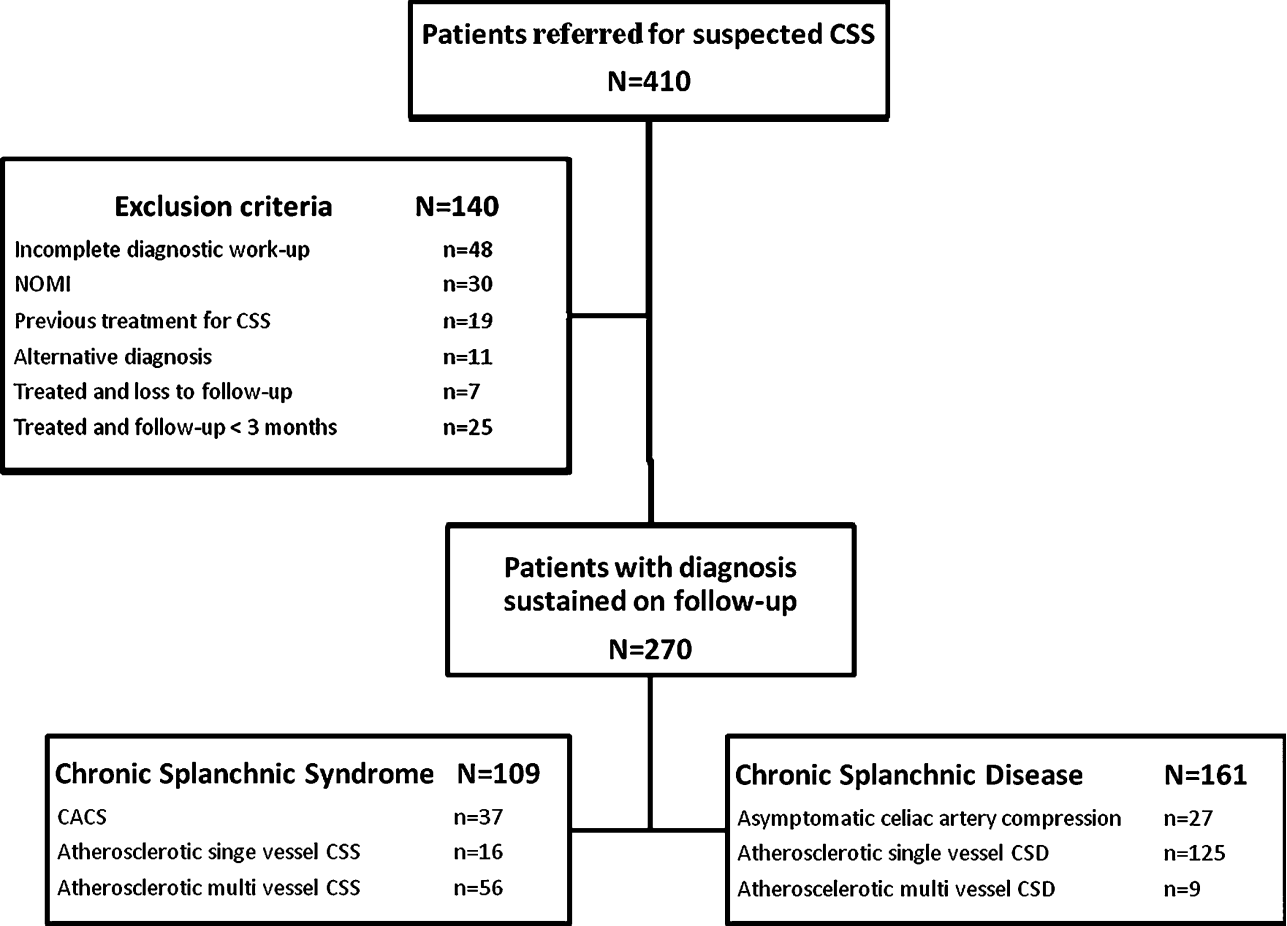
Flowchart of study protocol. *CSS* chronic splanchnic syndrome, *NOMI* nonobstructive mesenteric ischemia, *CACS* celiac artery compression syndrome, *CSD* chronic splanchnic disease

Treated patients were scheduled for follow-up. The first follow up appointment was 3 months after treatment, and thereafter every 6 months during the first 2 years for assessment of the medical history and a duplex ultrasound scan. Untreated patients were not seen on a regular basis in our center but were further treated by the referring physician. From a previous study, it was learned that this is a safe strategy [3]. Two patient groups were constructed based on their final diagnosis sustained on follow-up (Fig. 1).

### Statistical analysis

Data were analyzed with the Statistical Package of Social Sciences (SPSS Inc., Chicago, IL). Continuous data was expressed as mean (standard deviation) for normal variables and median (range) for non-normal variables. Nominal variables were expressed as the percentage of patients positive for this variable over the total number scored in the patient category.

Data between CSS and non-CSS patients were compared by using a χ^2^ test or Fisher’s exact test, as appropriate, for nominal variables, or Student’s *t* test or Wilcoxon’s rank-sum test, as appropriate, for continuous data. Relevant clinical features for the diagnosis of CSS were selected by a univariate analysis. Clinical features of CSS, significant at *p* < 0.15 in univariate analysis and scored in ≥100 patients were eligible for multivariate logistic regression analysis. For these factors, sensitivity, specificity, positive predictive value, and negative predictive value was calculated. *p* < 0.05 value was considered statistical significant. Nonsignificant variables were removed one by one from the full model, until the model significantly deteriorated, based on the −2 log likelihood. With these results, a predictive model was constructed and interaction between variables was tested if necessary. These data were expressed as odds ratio with 95% confidence interval (CI).

## Results

### Patient characteristics

During the study period, 410 patients were referred for analysis of suspected CSS. The flow chart of the study is presented in Fig. 1. A total of 270 patients with chronic abdominal symptoms and stenosis of at least one splanchnic artery were eligible for analysis; 109 patients had the final diagnosis CSS (72 with CSS and 37 with CACS), and 161 were diagnosed with CSD. Patient characteristics are outlined in Table 1.

**Table 1:** Characteristics of patients with final diagnosis CACS, CSS and CSD

Patient characteristics Total group (*n* = 270); 102 males; median age, 53 (range 15–84) years	Atherosclerotic CSS (*n* = 72)	CACS (*n* = 37)	CSD (*n* = 161)
Sex (male:female)	30:42*	6:31^#^	66:95
Median age (yr) (range)	65 (33–84)^$^*	34 (15–75)^#^	52 (16–84)
Mean body mass index (kg/m^2^) (SD)	22.5 (4.5)	21.8 (3.7)^#^	23.5 (4.6)
Diabetes	14%	0%^#^	11%
Hypertension	44%^$$^**	3%^##^	16%
Hypercholesterolemia	11%	6%	6%
Coronary artery disease	28%^$^*	0%^#^	14%
Peripheral artery disease	51%^$$^**	0%^##^	25%
Earlier episode of colonic ischemia	4%	3%	1%
Smoking	54%^$^	28%^#^	48%
Family members with atherosclerosis	74%^$^*	58%	57%
Median duration of follow-up (mo) (range)	10 (1–37)	15 (2–39)	8 (1–43)
Median duration of symptoms (mo) (range)	7.0 (0.2–120)	18 (1–252)	14 (0.1–408)
Patients without follow-up	6	0	122

*CSS* chronic splanchnic syndrome, *CACS* celiac artery compression syndrome, *CSD* chronic splanchnic disease

^$^ *p* < 0.05 for atherosclerotic CSS vs. no CSS; ^$$^ *p* < 0.001 for atherosclerotic CSS vs. CSD; * p < 0.05 for atherosclerotic CSS vs. CACS; ** *p* < 0.001 for atherosclerotic CSS vs. CACS; ^#^ *p* < 0.05 for CACS vs. CSD; ^##^ *p* < 0.001 for CACS vs. CSD

### CSS, CACS, and CSD

Overall clinical presentation of patients with CACS was comparable to patients with atherosclerotic CSS. For the CACS cohort, the duration of complaints was longer (18 vs. 7 months, *p* = 0.03), weight loss was less common in the CACS patients (72% vs. 91%, *p* = 0.01), and more patients with CACS experienced pain during stress (50% vs. 27%, *p* = 0.02). For further analysis, patients with CACS and atherosclerotic CSS were taken together and compared with CSD patients.

In patients with CSS, mean age was 54 (range, 16–84) years, and 33% was male. In the CSD, the mean age was 53 (range, 15–84) years, and 41% was male (not significant). Results from univariate analysis between CSS and CSD patients are presented in Table 2. The mean duration of symptoms was shorter in CSS vs. CSD (10.5 vs. 14.0 months, *p* = 0.02). Postprandial pain was significantly more often reported in CSS patients (87 vs. 72%, *p* = 0.007). Time between end of the meal and onset of the pain was not statistically significant between the two groups. Diarrhea and weight loss were significantly more often reported in patients with CSS compared with CSD (diarrhea 35% vs. 22%, *p* = 0.02; weight loss 85% vs. 70%, *p* = 0.006). The results of the univariate analysis of the clinical features are presented in Table 3. Their individual potential to distinguish CSS from CSD is low with the highest accuracy for diarrhea (61%) and the lowest accuracy for postprandial pain and change in eating habits (both 49%).

**Table 2:** Major features from medical history notes and questionnaire of CSS patients versus CSD patients; results from the univariate analysis

Clinical feature	Scored^b^	CSS (*n* = 109)	CSD (*n* = 161)	*p* value
Postprandial pain	228	87 %	71%	0.007^a^
Median interval end of meal – onset of pain (min) (range)	106	15 (5–120)	15 (5–360)	0.84
Median duration of pain (hr) (range)	113	2.0 (0.3–96)	2.0 (2.0–72)	0.09^a^
Weight loss	262	85%	70%	0.006^a^
Median weight loss in kg/month (range)	194	1.8 (0.1–12)	1.9 (0.3–14.3)	0.85
Adapted eating pattern^c^	168	90%	79%	0.07^a^
Smaller portions	129	100%	90%	0.02
Pain after exercise	262	62%	67%	0.45
Pain with stress	257	35%	41%	0.34
Pain in relation with posture	211	64%	56%	0.23
Diarrhea	264	35%	22%	0.02^a^

Total group (*n* = 270)

*CSS* chronic splanchnic syndrome; *CSD* chronic splanchnic disease

^a^Used for multivariate analysis; ^b ^number of patients in which the clinical feature was described; ^c ^smaller portions, change in meal composition

**Table 3:** Performance of significant clinical features to diagnose chronic splanchnic syndrome

Clinical feature	Sensitivity (%)	Specificity (%)	PPV (%)	NPV (%)	Accuracy (%)
Postprandial pain	72	35	41	66	49
Weight loss	80	30	44	75	52
Change in eating habits	90	21	44	75	49
diarrhea	34	78	49	65	61

*PPV* positive predictive value, *NPV* negative predictive value

### Risk stratification

The main clinical features with *p* < 0.15 in the univariate analysis and scored in >100 patients were used for multivariate logistic regression analysis. Adapted eating pattern (OR 3.1; 95% CI 1.08–8.88) and diarrhea (OR 2.6; 95% CI 1.31–5.3) were statistically significant clinical features in multivariate analysis. A prediction model was constructed from four significant of the clinical features in univariate analysis. These included “adapted eating pattern,” “weight loss,” “diarrhea,” and “postprandial pain.” This model could be tested in the 164 patients in whom the presence of absence of these factors was known (Table 4). The probability for CSS in absence of the four criteria was 13% (number needed to treat, 7.7). All four criteria were absent in seven patients, of whom one had multivessel CSS. The probability for CSS in presence of all four criteria was 60% (number needed to treat, 1.7). This was present in six patients in whom four actually had CSS. Two or three clinical features were present in the medical history of 151 patients.

**Table 4:** Probability of chronic splanchnic syndrome in the study population

Clinical features present in medical history	Probability of CSS (%)	NNT (mean)
0	13	7.7
1	14–29	7.1–3.4 (5.3)
2	18–51	5.5–2.0 (3.8)
3	37–51	2.7–2.0 (2.4)
4	60	1.7

The clinical features were adapted eating pattern, diarrhea, postprandial pain, and weight loss

*NNT* numbers needed to treat

## Discussion

The medical history of patients with splanchnic artery stenosis may help to predict the presence of chronic gastrointestinal ischemia (chronic splanchnic syndrome or CSS). In addition to the well-established ischemic complaints of pain after meals and weight loss, we found diarrhea and the presence of an adapted eating pattern as significant clinical features of CSS. In our study population, the likelihood for CSS increased from 13% when none of these four factors was present to 60% when all were present. Therefore, even in patients with the most suspicious clinical presentation of CSS, 40% of them where actually not suffering from their splanchnic artery stenosis (chronic splanchnic disease or CSD), and treatment would be not indicated.

Postprandial pain and the presence of weight loss were present in the majority of both CSS and CSD patients and did not help in differentiation between CSS and CSD. BMI and degree of weight loss was not statistically significant either. Probably this is all caused by selection bias because for most referring physicians, postprandial pain and weight loss trigger the suspicion of CSS. These are considered typical for GI ischemia [5]. The two complaints that were independent risk factors for the presence of CSS in this study, diarrhea and an adapted eating pattern, are less well established. Both factors have been described before in the clinical presentation of CSS as atypical features, but have not been appreciated as important factors [4].

The constructed model combining the two supposed classical risk factors (postprandial pain, weight loss) with adapted eating pattern and diarrhea had a modest performance in risk assessment of CSS. Furthermore, the performance to safely exclude low-risk patients from diagnostic workup was poor. In the absence of all four complaints, there was still a 13% probability on the presence of CSS. If this patient group would not undergo the diagnostic workup, this would lead to a minor reduction in workload, but at the expense of missing important pathology. The disappointing role of the medical history in ischemia detection is in line with a recent paper by Sana et al. who assessed the clinical features of CSS as well. Their strongest predictors were “postprandial pain” and “weight loss per kg/month.” Their data support our conclusion that clinical features alone have a limited value to correctly diagnose CSS [18].

The risk stratification model, as described in Table 4, may be used in daily clinical practice to estimate the risk for CSS if the severity of splanchnic artery stenosis is taken into consideration. Especially in patients with single vessel involvement, a low pre-test probability might justify a wait and see policy, because the prognosis on mortality is excellent and the risk on acute bowel infarction is absent. In multivessel disease, with a significant chance of bowel infarction and death, proper evaluation is indicated even in absence of ischemic complaints [19, 20]. Further studies, in nontertiary centres are needed to evaluate the utility of the constructed risk model.

Because clinical history alone is insufficient to discriminate between CSS and CSD, other diagnostic tests are needed. Both air tonometry and visible light spectroscopy are methods to detect mucosal ischemia in the gastrointestinal tract. The technical principles of both techniques have been extensively described elsewhere [2, 21]. Noord et al. showed that both tests have almost similar accuracy [21]. For tonometry, Otte et al. showed in a large patient cohort that sensitivity and specificity for detection of ischemia were 78% and 92%, respectively [11]. In a cohort with 28 patients ultimately diagnosed with CSS, 57% would have been missed with clinical history alone, whereas the accuracy of tonometry was 80% [13].

This analysis was performed from a large data set to identify factors in the clinical history that would help to preselect patients who would need further analysis, and hopefully eliminate individuals with a very low probability on CSS. The results of this study indicate that clinical history alone is clearly insufficient and a test that accurately demonstrates ischemia is needed.

This study has its methodological shortcomings. First, patients were included that were referred to our specialized working group. This has probably filtered out patients with less typical presentation for gastrointestinal ischemia. Probably, most patients with splanchnic artery stenosis are asymptomatic in daily life [22, 23]. Therefore, using the same four criteria in a nontertiary center will probably lead to even lower likelihoods for CSS. Second, the method of data collection has its drawbacks. To avoid missing data, we included both the notes of two highly experienced clinicians, as well as a standard questionnaire. The medical notes from the former contained more factors known to be specific for CSS and less nonspecific issues. Both clinicians only reported remarkable items in the medical history notes, either positive or negative, with regard to CSS as diagnosis. Parameters from the medical history notes could only be scored as present or absent when specifically mentioned. If it was not mentioned, it could not be ascertained with 100% certainty whether that item had been discussed with the patient. Therefore, it was scored as “missing.” This resulted in a reliable dataset, at the expense of loss of information. The data from the questionnaire were more complete on these less-typical complaints but may have been subject to interpretative problems.

In conclusion, in patients with splanchnic stenosis, four parameters determine the likelihood of having gastrointestinal ischemia. These factors are postprandial pain, weight loss, adapted eating pattern, and diarrhea. The likelihood rose from 13% to 60% when none or all were present. This is not sufficient to make a decision about which patient to treat. An accurate function test to demonstrate ischemia is needed for an accurate diagnosis of chronic gastrointestinal ischemia.

## Appendix

### Questionnaire

- Postprandial pain
  - After a meal, I experience
    1. No pain
    2. Pain, but it does not interfere with daily life
    3. Pain, with small impairment on daily life
    4. Pain, with severe impairment on daily life
    5. Pain, with total impairment on daily life
- Pain with exertion
  - After physical exercise, I experience
    1. No pain
    2. Pain, but it does not interfere with daily life
    3. Pain, with small impairment on daily life
    4. Pain, with severe impairment on daily life
    5. Pain, with total impairment on daily life
- Pain with stress
  - During mental stress, I experience
    1. No pain
    2. Pain, but it does not interfere with daily life
    3. Pain, with small impairment on daily life
    4. Pain, with severe impairment on daily life
    5. Pain, with total impairment on daily life
- Relationship with posture
  - Do you experience more pain in a certain posture?

Yes or no

- Stools
  - How many times a day do you defecate?
  - What is the consistency?
    1. Separate hard lumps, like nuts (hard to pass)
    2. Sausage-shaped, but lumpy
    3. Like peanut butter a sausage or snake, smooth and soft
    4. Watery, no solid pieces. Entirely liquid
- Weight loss
  - Did you lose weight recently?

Yes or no

- Degree of weight loss (kg/month)
  - I lost……kilograms of weight during the last……weeks / months / years
